# A Frequency-Based Approach for the Detection and Classification of Structural Changes Using *t*-SNE †

**DOI:** 10.3390/s19235097

**Published:** 2019-11-21

**Authors:** David Agis, Francesc Pozo

**Affiliations:** Control, Modeling, Identification and Applications (CoDAlab), Department of Mathematics, Escola d’Enginyeria de Barcelona Est (EEBE), Universitat Politècnica de Catalunya (UPC), Campus Diagonal-Besòs (CDB), Eduard Maristany, 16, 08019 Barcelona, Spain; david.agis@upc.edu

**Keywords:** classification detection, principal component analysis (PCA), structural changes, structural health monitoring (SHM), *t*-distributed stochastic neighbor embedding (*t*-SNE)

## Abstract

This work presents a structural health monitoring (SHM) approach for the detection and classification of structural changes. The proposed strategy is based on *t*-distributed stochastic neighbor embedding (*t*-SNE), a nonlinear procedure that is able to represent the local structure of high-dimensional data in a low-dimensional space. The steps of the detection and classification procedure are: (i) the data collected are scaled using mean-centered group scaling (MCGS); (ii) then principal component analysis (PCA) is applied to reduce the dimensionality of the data set; (iii) *t*-SNE is applied to represent the scaled and reduced data as points in a plane defining as many clusters as different structural states; and (iv) the current structure to be diagnosed will be associated with a cluster or structural state based on three strategies: (a) the smallest point-centroid distance; (b) majority voting; and (c) the sum of the inverse distances. The combination of PCA and *t*-SNE improves the quality of the clusters related to the structural states. The method is evaluated using experimental data from an aluminum plate with four piezoelectric transducers (PZTs). Results are illustrated in frequency domain, and they manifest the high classification accuracy and the strong performance of this method.

## 1. Introduction

Structural health monitoring (SHM) is a crucial process for engineering structures because it checks the correct behavior of the structure and determines whether it needs some type of maintenance. The healthy state of the structure has to remain between the specified limits or threshold, but these limits may change due to the aging of the structure and its use, or due to the environmental and operational conditions (EOC). Hence, in SHM systems, detection and classification of structural changes are essential in order to know the current state of the structure for security and to reduce costs of inspection and maintenance. If damage is detected and classified precisely at the time it occurs, some action may be taken before a human and/or economic disaster occurs, thus reducing the probability of accidents and the maintenance costs. SHM has been applied in many structures such as wind turbines [1,2,3], buildings [4,5], and aircraft [6,7], among others, and a review of the state-of-the-art manifests that SHM is a very active research field.

With the goal of obtaining information about the state of the structure, data are collected by a sensor network, which is placed along the structure. The information obtained from multi-sensor signals creates a high-dimensional data set with a large volume of data due to continuous measurements of the monitoring system. Various methods have been proposed for the handling of high-dimensional, big, and complex data. Among these methods, plane or spatial representation techniques stick out as they offer a way to handle this type of data by means of an interface that allows an easy detection of natural clusters, identifying hidden patterns, et cetera [8]. Plane or spatial representation techniques are also somehow related to dimensionality reduction. Dimensionality reduction is the mechanism of reducing the dimension of the original data, while keeping mostly the same intrinsic information [9]. One of the proposed dimensionality reduction methods in the literature is *t*-distributed stochastic neighbor embedding (*t*-SNE), a technique developed by L. van der Maaten and G. Hinton [10], which is able to represent the local structure of original high-dimensional data in a low-dimensional space (for example, a simple 2-D plot). This technique detects patterns by identifying clusters based on similarity of data points. *t*-SNE is widely used in the literature as a dimensionality reduction technique, as a classification or pattern recognition method, or as a visualization and compression method of big data sets, but although *t*-SNE has been applied in several applications, this is one of the first approaches of *t*-SNE in the field of SHM [11].

In the present approach, *t*-SNE is applied in the frequency domain. In the field of SHM and condition monitoring (CM) this is sometimes common, and the combination of time-frequency domain is also used. Some examples are Tsogka et al. [12], who propose a novel vibration-based SHM method for damage detection in the frequency domain, which illustrates its practical application in the case of a historic bell tower. Xu et al. [13] propose a clustering method based on ensemble empirical mode decomposition and affinity propagation for bearing performance degradation assessment. To prove the superiority of the approach, the proposed methodology is compared to various popular clustering methods and commonly used time-domain indicators. The results show that the proposed method outperforms these popular clustering methods and time-domain indicators. Cheng et al. [14] propose a multisensory data-driven health degradation monitoring system by using a generalized multiclass support vector machine. In this method, multidimensional feature extraction is implemented in the time domain, frequency domain, and time-frequency domain.

In this work, a SHM strategy for detection and classification of structural changes based on a two-step data integration (type *E* unfolding [15] and the so-called mean-centered group scaling (MCGS)), data transformation using PCA, and a two-step data reduction combining PCA and *t*-SNE has been proposed. PCA is an extensively used technique that is mainly used for dimensionality reduction or feature extraction in the framework of pattern recognition [16], and it can be applied differently to detect and classify structural changes or faults [17]. In some cases, however, it can be observed that the projection into the first principal components does not allow a visual grouping, clustering, or separation. For this reason, we propose the damage or fault detection based on the combination of PCA and *t*-SNE. As a consequence, the basic steps of the detection and classification procedure that we apply are: (i) the data collected, in the frequency domain, are first scaled using MCGS due to the different scales and magnitudes in the measurements; (ii) then PCA is applied to obtain a better representation of the original data, by reducing the dimensionality of the scaled data and projecting the scaled data into the vectorial space spanned by the principal components; and (iii) *t*-SNE is finally applied to the projected data to represent these points as points in a plane. It will be shown that, with respect to the time domain, the quality of the clusters related to the different structural states is significantly improved. More precisely, the current structure to be diagnosed will then be associated with a structural state based on three different strategies: (i) the smallest point-centroid distance, i.e., when a single actuation phase is considered; (ii) majority voting; and (iii) sum of the inverse distances, i.e., when several actuation phases are combined. Therefore, in this work, *t*-SNE is used, in combination with a particular data integration, data transformation, and data reduction, for the first time in the field of SHM in a frequency-based approach. In comparison to previous strategies found in the literature, this novel method is able to yield a best detection and classification of structural changes, thus leading to a best performance.

The proposed method for the detection and classification of structural changes is assessed using experimental data from a plate with four piezoelectric transducers (PZTs). Since guided wave propagation-based SHM strategies have proven their ability to adequately identify defects in structures [18,19,20,21], in the present work, we have also considered the paradigm of guided waves. In this paradigm, the structure is excited by a signal and the response is measured to create a baseline pattern. When a new structure has to be diagnosed, it has to be excited by the exact same signal and the response is measured and compared with the baseline pattern. Results reveal the high classification accuracy and the strong performance of this methodology, with a percentage of correct decisions of about 100% in various scenarios. In the present work, the environmental conditions were not considered, as it will be the topic for further developments.

The structure of the paper is as follows: Section 2 describes the objective of *t*-SNE and how the plane or spatial representation is obtained. Section 3 includes how the baseline data are collected and pre-processed, how the global dimension of the data is reduced, and how the clusters are created using *t*-SNE. The damage detection and classification procedure of a structure that has to be diagnosed is presented in Section 4. The experimental case study is described in Section 5. In Section 6, the results are shown. Finally, in Section 7, some conclusions are drawn.

## 2. *t*-Distributed Stochastic Neighbor Embedding (*t*-SNE)

### 2.1. The Objective of t-SNE

*t*-SNE is an improved variation of the technique so-called stochastic neighbor embedding (SNE) [22]. With respect to SNE, *t*-SNE is much easier to optimize and yields better plane or spatial representations of the high-dimensional data, since it reduces the tendency to crowd points in the center of the distribution (the so-called *crowding problem*). Part of the enhancements of *t*-SNE with respect to SNE are due to the fact that the cost function used by *t*-SNE differs from the one used by SNE in two features: (i) *t*-SNE uses a symmetrized version of the SNE cost function with simpler gradients; and (ii) *t*-SNE uses a Student’s *t*-distribution, instead of a Gaussian, to calculate the similarity between two points in the low-dimensional space.

Given a collection of high-dimensional data points:

(1) $$\begin{array}{c} X=\left\{x^{1},\ldots ,x^{\nu }\right\}\subset R^{D},\;\nu ,D\in N, \end{array}$$

the aim is to find a collection of low-dimensional map points

$$\begin{array}{c} Y=\left\{y^{1},\ldots ,y^{\nu }\right\}\subset R^{d},\;d\in N \end{array}$$

that form a faithful representation of the original points $\left\{x^{1},\ldots ,x^{\nu }\right\}$ in a lower-dimensional space. Typical values for *d* are 2 (*plane* representation) or 3 (*spatial* representation), where d≪D. By saying *faithful* representation, we mean that the points $\left\{y^{1},\ldots ,y^{\nu }\right\}$ in the lower-dimensional space preserve, as much as possible, the local structure of the original data X.

### 2.2. Pairwise Similarities

To preserve local similarities of the original data X by this embedding, *t*-SNE first converts the high-dimensional Euclidean distances between data points $x^{i}$ and $x^{j}$ into conditional probabilities by centering a Gaussian distribution at $x^{i}$, computing the density of $x^{j}$ under this Gaussian distribution, and renormalizing:

(2) $$\begin{array}{c} p_{j|i}=\frac{\exp\left(\frac{-∥x^{i}-x^{j}{∥}_{2}^{2}}{2\sigma_{i}^{2}}\right)}{\sum_{\begin{array}{c} l=1\\ l\neq i \end{array}}^{\nu }\exp\left(\frac{-∥x^{i}-x^{l}{∥}_{2}^{2}}{2\sigma_{i}^{2}}\right)},\;i,j=1,\ldots ,\nu ,\;i\neq j, \end{array}$$

where $\frac{∥x^{i}-x^{j}{∥}_{2}^{2}}{2\sigma_{i}^{2}}$ (*affinity or scaled squared Euclidean distance*) is the dissimilarity between data points $x^{i}$ and $x^{j}$. The variance of the Gaussian distribution, $\sigma_{i}^{2}$, is computed automatically. Since only pairwise similarities between data points are of interest, *t*-SNE imposes $p_{i|i}=0$. This conditional probability measures the similarity of $x^{j}$ to $x^{i}$. If two data points are close, $p_{j|i}$ will be large. However, if two data points are far, $p_{j|i}$ will be small.

Then, by symmetrizing the conditional probability in Equation (2), the joint probability is defined as follows:

$$\begin{array}{cc} p_{ij} & =\frac{p_{j|i}+p_{i|j}}{2\nu },\;i,j=1,\ldots ,\nu ,\;i\neq j,\\ p_{ii} & =0. \end{array}$$

The joint probability also measures the pairwise similarity between data points $x^{i}$ and $x^{j}$. As a result, let us define the similarity matrix $P\in M_{\nu \times \nu }\left(R\right)$ for the high-dimensional data points as $P={\left(p_{ij}\right)}_{i,j=1,\ldots ,\nu }$.

When the similarity matrix for the data points X in Equation (1) is obtained, *t*-SNE also defines the similarity matrix $Q\in M_{\nu \times \nu }\left(R\right)$ for the map points Y. Essentially, we build matrix Q following the same idea as for the similarity matrix P with respect to original data points. The one and only difference is that we use for matrix Q a renormalized Student’s *t*-distribution with one degree of freedom and $\sigma_{i}^{2}=\frac{1}{2}$ for all *i*, instead of a Gaussian distribution:

(3) $$\begin{array}{cc} q_{ij} & =\frac{\frac{1}{1+∥y^{i}-y^{j}{∥}_{2}^{2}}}{\sum_{k=1}^{\nu }\sum_{\begin{array}{c} l=1\\ l\neq k \end{array}}^{\nu }\frac{1}{1+∥y^{k}-y^{l}{∥}_{2}^{2}}},\;i,j=1,\ldots ,\nu ,\;i\neq j, \end{array}$$

(4) $$\begin{array}{cc} q_{ii} & =0, \end{array}$$

where $q_{ij}$ represents the local structure of the data points in the low-dimensional space.

### 2.3. Comparing Similarity Matrices: Cost Function

The goal is to select the map points so that the two similarity matrices, P and Q, are as similar as possible. The similarity between these two matrices will be defined in terms of the Kullback–Leibler (KL) divergence. The KL divergence between the joint probability distributions P and Q is a measure of the *distance* between the two similarity matrices, and it can be defined as [10,22,23]:

(5) $$\begin{array}{c} C=D_{KL}\left(P∥Q\right)=\sum_{i=1}^{\nu }\sum_{\begin{array}{c} j=1\\ j\neq i \end{array}}^{\nu }p_{ij}\log\left(\frac{p_{ij}}{q_{ij}}\right). \end{array}$$

Therefore, minimizing the KL divergence reduces the *distance* between these two matrices. And to minimize the cost function C, the gradient descent method is used: $\frac{\partial C}{\partial y^{i}}$. It is worth noting that the gradient descent is an iterative optimization algorithm and therefore it updates the map point $y^{i}$ at each step.

For more details, see the original *t*-SNE paper [10].

## 3. Data Collection, Pre-Processing, and Clustering: Baseline Data

### 3.1. Data Collection and Pre-Processing

The data collected are made up of different response signals measured, in the time domain, by sensors on a vibrating structure. Multiple realizations of these responses are measured, under different structural states. Coming up next, these responses signals are transformed into the frequency domain using the fast Fourier transform (FFT) algorithm, and features are extracted from the spectrum to reduce the data dimension, dividing by two and adding one to the number of components in each signal. A matrix which collects all the realizations under different structural states in the frequency domain is defined as:

(6) $$\begin{array}{cc} X=\left(x_{i,l}^{k,j}\right) & =\left[\begin{array}{ccccccc} x_{1,1}^{1,1} & \cdots & x_{1,1}^{1,L} & \cdots & x_{1,1}^{N,1} & \cdots & x_{1,1}^{N,L}\\ \vdots & \ddots & \vdots & \ddots & \vdots & \ddots & \vdots \\ x_{n_{1},1}^{1,1} & \cdots & x_{n_{1},1}^{1,L} & \cdots & x_{n_{1},1}^{N,1} & \cdots & x_{n_{1},1}^{N,L}\\ x_{1,2}^{1,1} & \cdots & x_{1,2}^{1,L} & \cdots & x_{1,2}^{N,1} & \cdots & x_{1,2}^{N,L}\\ \vdots & \ddots & \vdots & \ddots & \vdots & \ddots & \vdots \\ x_{n_{2},2}^{1,1} & \cdots & x_{n_{2},2}^{1,L} & \cdots & x_{n_{2},2}^{N,1} & \cdots & x_{n_{2},2}^{N,L}\\ \vdots & \ddots & \vdots & \ddots & \vdots & \ddots & \vdots \\ x_{1,E}^{1,1} & \cdots & x_{1,E}^{1,L} & \cdots & x_{1,E}^{N,1} & \cdots & x_{1,E}^{N,L}\\ \vdots & \ddots & \vdots & \ddots & \vdots & \ddots & \vdots \\ x_{n_{E},E}^{1,1} & \cdots & x_{n_{E},E}^{1,L} & \cdots & x_{n_{E},E}^{N,1} & \cdots & x_{n_{E},E}^{N,L} \end{array}\right] \end{array}$$

(7) $$\begin{array}{cc} \; & =\left[\begin{array}{c} X_{1}\\ X_{2}\\ \vdots \\ X_{E} \end{array}\right]\in M_{\left(n_{1}+\cdots +n_{E}\right)\times \left(N\cdot L\right)}\left(R\right), \end{array}$$

(8) $$\begin{array}{c} =\left[\begin{array}{cccc} X^{1} & X^{2} & \cdots & X^{N} \end{array}\right], \end{array}$$

where N∈N is the number of sensors and k=1,…,N identifies the sensor that is measuring; L∈N is the number of components in each signal and j=1,…,L indicates the *j*-th measurement in the frequency domain; E∈N is the number of different structural states that are considered and l=1,…,E represents the structural state that is been measured; and finally, $n_{l},\;l=1,\ldots ,E$, is the number of realizations per structural state and $i=1,\ldots ,n_{l}$ is the *i*-th realization related to the *l*-th structural state. Note that matrix X in Equation (7) is formed by *E* horizontal blocks, $X_{l},\;l=1,\ldots ,E$, each one of them related to the different structural states. At the same time, this matrix X can also be viewed as formed by *N* vertical blocks, $X^{k},\;k=1,\ldots ,N$, each one of them related to the different sensors. Due to the different scales and magnitudes in the measurements, the matrix X in Equation (7) is rescaled using MCGS, which is suggested by Pozo et al. [24].

### 3.2. Dimensionality Reduction

One of the main reasons of using the MCGS is that the covariance matrix of matrix $\overset{˘}{X}$, i.e., the scaled data set, can be computed in a very simple way as:

(9) $$\begin{array}{c} C_{\overset{˘}{X}}=\frac{1}{n-1}{\overset{˘}{X}}^{⊤}\overset{˘}{X}\in M_{\left(N\cdot L)\times (N\cdot L\right)}\left(R\right). \end{array}$$

The eigenvectors $\rho_{k},\;k=1,\ldots ,N\cdot L,$ and eigenvalues $\lambda_{k},\;k=1,\ldots ,N\cdot L,$ of the covariance matrix $C_{\overset{˘}{X}}$ define the subspaces in the PCA model. The eigenvalues $\lambda_{k}$ are then ordered in decreasing order as

$$\begin{array}{c} \lambda_{1}\geq \lambda_{2}\geq \cdots \geq \lambda_{N\cdot L}, \end{array}$$

and the matrix $P\in M_{\left(N\cdot L)\times (N\cdot L\right)}\left(R\right)$ contains, written as columns, their corresponding eigenvectors $\rho_{k}$. These eigenvectors are known as the principal components. The eigenvalues define the partial variance of each eigenvector. When the *column scaling* is applied to matrix X in Equation (7), although not not the case of this paper, we have that the trace of the covariance matrix $C_{\overset{˘}{X}}$, which is the sum of the eigenvalues, is equal to the number of columns of X, that is, N·L. This means that the proportion of the variance directed along the first ℓ∈N principal components is given by $\frac{\lambda_{1}+\cdots +\lambda_{\ell }}{N\cdot L}$. However, when the MCGS is applied to scale the raw data in matrix X in Equation (7), the trace of the covariance matrix $C_{\overset{˘}{X}}$ is no longer necessarily equal to N·L. As a consequence, the proportion of the variance explained by the first *ℓ* principal components is given by:

$$\begin{array}{c} \frac{\lambda_{1}+\cdots +\lambda_{\ell }}{\lambda_{1}+\cdots +\lambda_{N\cdot L}}. \end{array}$$

In this work, we use PCA to reduce the dimensionality of the scaled data set $\overset{˘}{X}$ by selecting a reduced, but still significant, number ℓ=D<N·L of principal components. This dimensionality reduction is performed through the reduced matrix

(10) $$\begin{array}{c} P_{\ell }=P_{D}=\left[\begin{array}{cccc} \rho_{1} & \rho_{2} & \cdots & \rho_{\ell } \end{array}\right]\in M_{(N\cdot L)\times \ell }\left(R\right), \end{array}$$

which is composed by the concatenation of the eigenvectors $\rho_{k}$ related to the highest eigenvalues. Matrix $P_{\ell }=P_{D}$ in Equation (10) is the *model* or *PCA model*. The scaled data set $\overset{˘}{X}$ is then projected into the vectorial space spanned by the ℓ=D first principal components through the premultiplication of $P_{\ell }$ by $\overset{˘}{X}$. More precisely,

(11) $$\begin{array}{c} T_{\ell }=T_{D}=\overset{˘}{X}P_{\ell }\in M_{n\times \ell }\left(R\right). \end{array}$$

$P_{\ell }$ in Equation (10) has been defined as the PCA model that includes multiple realizations under different structural states. At the same time, $T_{\ell }$ in Equation (11) is the projection of the scaled data set $\overset{˘}{X}$ into the subspace spanned by the PCA model. The number of principal components ℓ=D∈N is chosen so that the proportion of the variance explained is greater than or equal to 95%.

### 3.3. Clustering Effect

In Section 3.2, the dimensionality reduction has been performed. More precisely, *n* realizations under different structural states (the rows of matrix X in Equation (7)), that may be seen as N·L−dimensional vectors, are projected and transformed into ℓ=D−dimensional vectors. This reduction of the dimension of the original data is performed with a small loss of information, less than 5%, and it is also expected that *ℓ* is much smaller than N·L.

A second transformation is performed to the projected data in matrix $T_{\ell }$ in Equation (11) using the *t*-SNE presented in Section 2. Let us define

$$\begin{array}{l} x^{i}=e_{i}^{⊤}T_{\ell }=e_{i}^{⊤}\overset{˘}{X}P_{\ell }\in R^{\ell },\;i=1,\ldots ,n \end{array}$$

as the *i*-th row of matrix $T_{\ell }$ in Equation (11). The vector $e_{i}\in R^{n}$ is the *i*-th element of the canonical basis. Let us also define

(12) $$\begin{array}{l} X=\left\{x^{1},\ldots ,x^{n}\right\}\subset R^{\ell } \end{array}$$

as a collection of high-dimensional data points. The objective is to find a collection of 2−dimensional map points

$$\begin{array}{c} Y=\left\{y^{1},\ldots ,y^{n}\right\}\subset R^{2} \end{array}$$

that represent the original set X with no explicit loss of information and preserving the local structure of this set. After the application of *t*-SNE, we expect *E* clusters to be observed, related to the *E* different structural states. These clusters are formed by the map points:

(13) $$\begin{array}{cc} \left\{y^{1},\ldots ,y^{n_{1}}\right\} & \subset Y,\mathrm{related}\;\mathrm{to}\;\mathrm{the}\;1\mathrm{st}\;\mathrm{structural}\;\mathrm{state};\\ \left\{y^{n_{1}+1},\ldots ,y^{n_{1}+n_{2}}\right\} & \subset Y,\mathrm{related}\;\mathrm{to}\;\mathrm{the}\;2\mathrm{nd}\;\mathrm{structural}\;\mathrm{state};\\ \left\{y^{n_{1}+n_{2}+1},\ldots ,y^{n_{1}+n_{2}+n_{3}}\right\} & \subset Y,\mathrm{related}\;\mathrm{to}\;\mathrm{the}\;3rd\;\mathrm{structural}\;\mathrm{state};\\ \; & \;\;\vdots \\ \left\{y^{n-n_{E-1}-n_{E}+1},\ldots ,y^{n-n_{E}}\right\} & \subset Y,\mathrm{related}\;\mathrm{to}\;\mathrm{the}\;\mathrm{penultimate}\;\mathrm{structural}\;\mathrm{state};\mathrm{and}\\ \left\{y^{n-n_{E}+1},\ldots ,y^{n}\right\} & \subset Y,\mathrm{related}\;\mathrm{to}\;\mathrm{the}\;\mathrm{last}\;\mathrm{structural}\;\mathrm{state}. \end{array}$$

## 4. Damage Detection and Classification Procedure: Structure to Diagnose

In Section 3.3, we have seen how the original realizations under different structural states are finally projected on a plane to define a set of clusters. In this section, we will present the damage detection and classification procedure of a structure that has to be diagnosed.

A single realization of the current structure to diagnose is needed. The data collected are made up, in this case, of different response signals measured by the same number of sensors *N* and the same number of components in each signal *L*, as in Equation (7). When these measures are obtained and they are transformed into the frequency domain, a new data vector z is constructed:

$$\begin{array}{c} z^{⊤}=\left[\begin{array}{ccccccc} z^{1,1} & \cdots & z^{1,L} & \cdots & z^{N,1} & \cdots & z^{N,L} \end{array}\right]\in R^{N\cdot L}. \end{array}$$

### 4.1. Scaling (MCGS)

Before the collected data coming from the structure to diagnose is projected into the space spanned by the principal components, the row vector $z^{⊤}$ has to be scaled to define a scaled row vector ${\overset{˘}{z}}^{⊤}$:

(14) $$\begin{array}{c} {\overset{˘}{z}}^{k,j}=\frac{z^{k,j}-\mu^{k,j}}{\sigma^{k}},\;k=1,\ldots ,N,\;j=1,\ldots ,L, \end{array}$$

where $\mu^{k,j}$ is the arithmetic mean of all the elements in the [(k−1)L+j]-th column of matrix X in Equation (6) i.e., the *j*-th column of the vertical block $X^{k}$ in Equation (8); and $\sigma^{k}$ is the standard deviation of all the elements in the vertical block $X^{k}$ in Equation (8) with respect to the mean value $\mu^{k}$ (the arithmetic mean of all the elements in the vertical block $X^{k}$ in Equation (8)).

### 4.2. Projection (PCA)

The projection of the scaled row vector ${\overset{˘}{z}}^{⊤}\in R^{N\cdot L}$ into the space spanned by the *ℓ* first principal components in $P_{\ell }$ is performed through the following vector to matrix multiplication:

$$\begin{array}{c} x^{n+1}={\overset{˘}{z}}^{⊤}\cdot P_{\ell }\in R^{\ell }. \end{array}$$

It is worth noting that the N·L−dimensional vector that contains the collected data coming from the structure to be diagnosed in now transformed into an ℓ−dimensional vector. This new point will be added to the data set X in Equation (12) to define a new set:

(15) $$\begin{array}{c} X^{\prime }=X\cup \left\{x^{n+1}\right\}=\left\{x^{1},\ldots ,x^{n},x^{n+1}\right\}\subset R^{\ell }. \end{array}$$

### 4.3. t-SNE and Final Classification

*t*-SNE is applied to the ℓ−dimensional data set $X^{\prime }$ in Equation (15) to find a collection of 2−dimensional map points:

$$\begin{array}{c} Y^{\prime }=\left\{y^{1},\ldots ,y^{n},y^{n+1}\right\}\subset R^{2} \end{array}$$

that represent the original set X with no explicit loss of information and preserving the local structure of this set, as well as including the map point $y^{n+1}$ associated to the data point $x^{n+1}$. The same *E* clusters have to be observed, related to the *E* different structural states. As in Section 3.3, these clusters are formed by the map points in Equation (13).

For each cluster, we compute its centroid, that is, the mean of the values of the points of data in the cluster. For instance, the centroid associated with the first structural state is:

$$\begin{array}{c} Y_{1}:=\frac{1}{n_{1}}\sum_{i=1}^{n_{1}}y^{i}=\frac{y^{1}+\cdots +y^{n_{1}}}{n_{1}}\in R^{2}, \end{array}$$

whereas the centroid associated with the second structural state is:

$$\begin{array}{c} Y_{2}:=\frac{1}{n_{2}}\sum_{i=1}^{n_{2}}y^{n_{1}+i}=\frac{y^{n_{1}+1}+\cdots +y^{n_{1}+n_{2}}}{n_{2}}\in R^{2}. \end{array}$$

**Figure 1:**
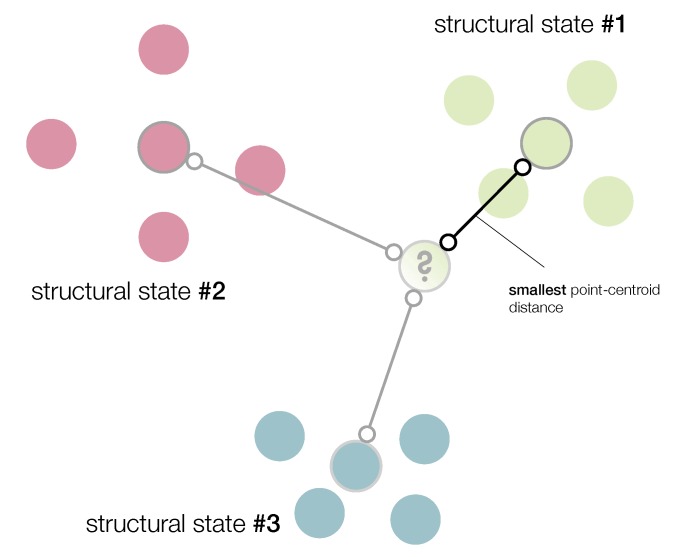
The current structure to diagnose is associated to the structural state with the smallest point-centroid distance.

In general, the centroid associated with the *l*-th structural state, l=1,…,E, is the point of the plane defined as

(16) $$\begin{array}{c} Y_{l}:=\frac{1}{n_{l}}\sum_{i=1}^{n_{l}}y^{\left(\sum_{j=0}^{l-1}n_{j}\right)+i}\in R^{2},\;l=1,\ldots ,E, \end{array}$$

where $n_{0}=0$. Therefore, the current structure to diagnose is associated to the *l*-th structural state if

$$\begin{array}{c} l=\arg\min_{l=1,\ldots ,E}{∥Y_{l}-y^{n+1}∥}_{2}, \end{array}$$

that is, if the minimum distance between $y^{n+1}$ and each one of the centroids corresponds to the Euclidean distance between $y^{n+1}$ and $Y_{l}$. We call this approach the smallest point-centroid distance (see Figure 1).

**Figure 2:**
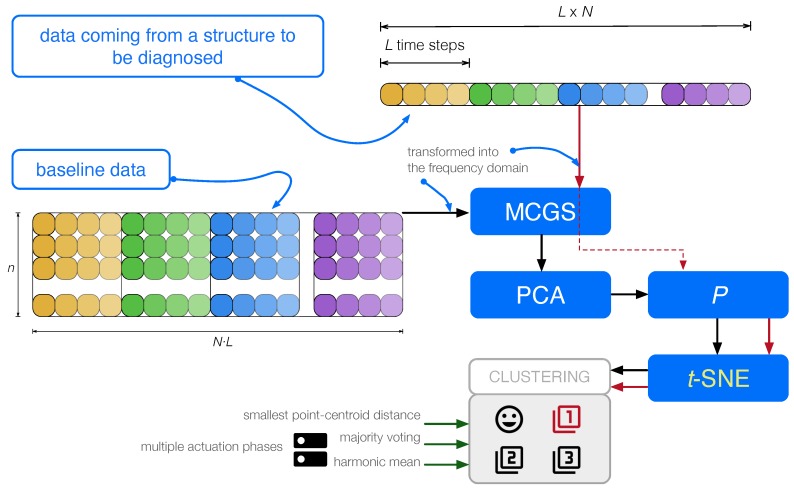
Flowchart of the proposed approach. Data coming from a structure are first transformed into the frequency domain and scaled, and then projected into the principal component analysis (PCA) model. Finally, *t*-distributed stochastic neighbor embedding (*t*-SNE) is used to create the clusters that will be used in the detection and classification of structural changes. MCGS = mean-centered group scaling.

A flowchart of the proposed approach and how it is applied is given in Figure 2.

## 5. Case Study: Aluminum Plate with Four PZTs

### 5.1. Structure

**Figure 3:**
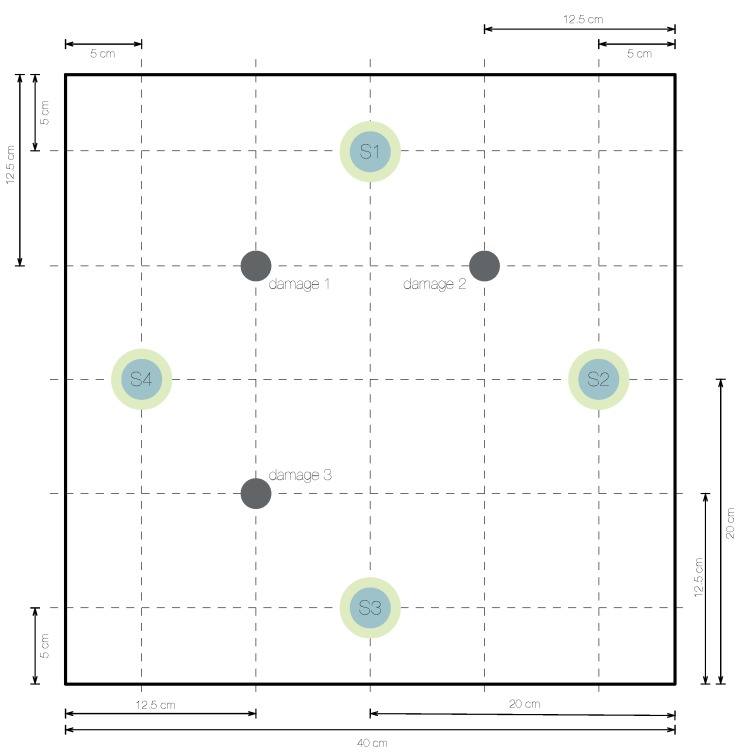
Aluminum plate instrumented with four piezoelectric sensors (S1,S2,S3, and S4).

In this section, a square aluminum plate with an area of 1600 cm${}^{2}$ (40 cm ×40 cm, and a thickness of 0.2 cm) and instrumented with four PZTs is considered to demonstrate the reliability of the damage detection and classification methodology introduced in Section 3 and Section 4. The piezoelectric transducer discs are attached to the surface and their location is shown in Figure 3. Assuming that the lower left corner of the plate in Figure 3 represents the origin of coordinates, the PZTs are installed at these positions (units in centimeters):

- PZT1 at (20,35)
- PZT2 at (35,20)
- PZT3 at (20,5)
- PZT4 at (5,20)

**Figure 4:**
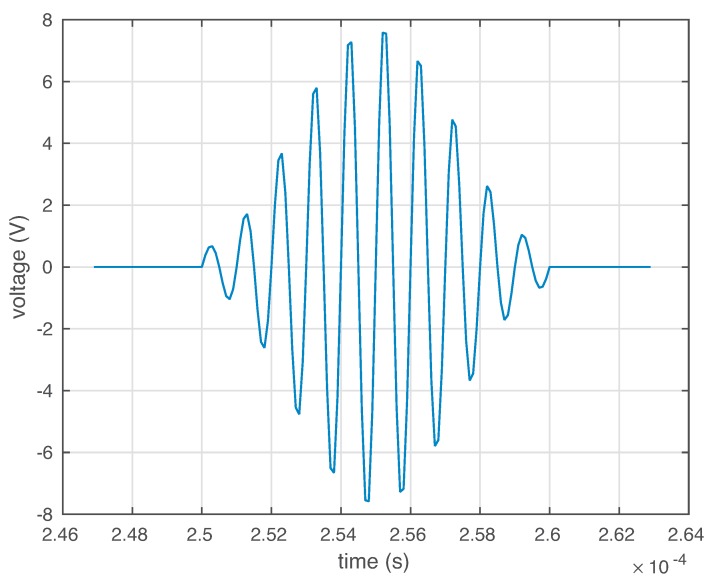
In actuator mode, this burst signal is applied to the piezoelectric transducers (PZTs) to produce a mechanical vibration.

These PZTs are able to work both in actuator mode and in sensor mode. In actuator mode, the burst signal in Figure 4 is applied to the PZTs, and they produce a mechanical vibration; and in sensor mode, they detect time varying mechanical response. It is worth keeping in mind that the distance between the four sensors is not the same. More precisely, for example, the distance between sensor 1 and sensor 2 and the distance between sensor 1 and sensor 4 is equal. However, the distance between sensor 1 and sensor 3 is relatively larger.

A 17.2916 grams mass is added to simulate the damage, in a non-destructive way, in the aluminum plate. This mass is an attached magnet in both sides of the plate, since aluminum is non-magnetic metal. This kind of damage is used to change the properties of the structure and to produce changes in the propagated wave, therefore providing different scenarios for validating the proposed method. The location of the mass defines each damage. These locations are (units in centimeters):

- damage 1 at (12.5,27.5)
- damage 2 at (27.5,27.5)
- damage 3 at (12.5,12.5)
  E=4 structural states are considered here:
- the first structural state corresponds to the healthy state of the structure, that is, the square aluminum plate with no damage;
- the second, third, and fourth structural states correspond to the plate with an added mass at the positions indicated in Figure 3 as damage 1, damage 2, and damage 3, respectively.

**Figure 5:**
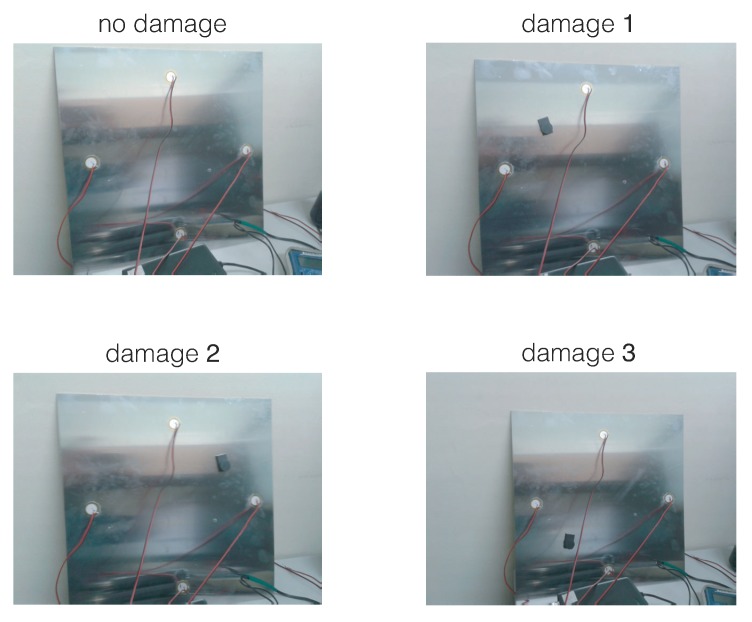
Aluminum plate with four PZTs and with four different structural states.

The aluminum plate is isolated from the vibration and noise that could affect the laboratory, as can be observed in Figure 5.

### 5.2. Scenarios and Actuation Phases

The experimental setup includes three different scenarios to determine the behavior of the methodology under the presence of white Gaussian noise, filters, and with respect to the length of the wire that is used from the digitizer to the sensors:

- **Scenario 1**. The signals are obtained using a short wire (0.5 m) from the digitizer to the PZTs, and these signals are filtered with a Savitzky–Golay (SG) [25] filter algorithm after adding white Gaussian noise. The filter is applied for the intention of smoothing the data.
- **Scenario 2**. The signals are obtained using a short wire (0.5 m) from the digitizer to the PZTs, but these signals are not filtered.
- **Scenario 3**. The signals are obtained using a long wire (2.5 m) from the digitizers to the PZTs. Signals are also filtered with the SG algorithm.

In this manner, we can observe the effect of the attenuation with short and long wires, the effect of adding white Gaussian noise to the measured signals, and the effect of the use of a SG filter in the detection and classification procedure.

As stated in Section 5.1, four PZTs (PZT1, PZT2, PZT3, and PZT4) are used to excite the aluminum plate and collect the measured response. This sensor network works in what we call *actuation phases*. In each actuation phase, a single PZT is used as an actuator (active sensor: the PZT excites the structure with a given excitation signal), and the rest of the PZTs are used as sensors (passive sensors: PZTs measure signals). Therefore, we have as many *actuation phases* as *sensors*:

- **Actuation phase 1**. PZT1 is used as the actuator, and PZT2, PZT3, and PZT4 are used as sensors.
- **Actuation phase 2**. PZT2 is used as the actuator, and PZT1, PZT3, and PZT4 are used as sensors.
- **Actuation phase 3**. PZT3 is used as the actuator, and PZT1, PZT2, and PZT4 are used as sensors.
- **Actuation phase 4**. PZT4 is used as the actuator, and PZT1, PZT2, and PZT3 are used as sensors.

It is very common in the literature, when using a sensor data fusion as in J. Vitola et al. [26,27], to merge the data that come from the different actuation phases in a single data matrix. In this paper, the approach with a single data matrix is also considered, but the case where each actuation phase is used as a classifier is additionally examined in Section 5.5.

### 5.3. Data Collection

Given a particular scenario, as the three defined in Section 5.2, four matrices X[φ],φ=1,…,4, one for each actuation phase, are obtained. Each matrix X[φ],φ=1,…,4, is organized as follows:

- $n_{1}=n_{2}=n_{3}=n_{4}=25$ experiments or realizations are performed for each structural state. Consequently, each matrix X[φ],φ=1,…,4, consists of 100 rows, that is $n_{1}+n_{2}+n_{3}+n_{4}=25\cdot 4$. More precisely, the first 25 rows represent the structure with no damage, the next 25 are realization where damage 1 is present in the structure, and so on.
- For each actuation phase φ,φ=1,…,4, we measure N=3 PZTs working as sensors during 60000 time instants. Then, these measurements are transformed into the frequency domain. Therefore, the number of columns of matrix X[φ],φ=1,…,4, is equal to N·L=3·((60000/2)+1)=90003.

Therefore, the matrix that collects all the realizations under the four different structural states in the frequency domain is (see Equation (6): here L=30001 and E=4):

(17) $$\begin{array}{cc} \; & X\left[\varphi \right]=\left(x{\left[\varphi \right]}_{i,l}^{k,j}\right)\in M_{100\times 90003}\left(R\right). \end{array}$$

The damage detection and classification procedure introduced in Section 3 and Section 4 can be applied to each one of the matrices X[φ],φ=1,…,4, in Equation (17), thus leading to one classification per actuation phase. But we can also use the horizontal concatenation of the four matrices X[φ],φ=1,…,4, to obtain the matrix:

(18) $$\begin{array}{c} X\left[1,2,3,4\right]=\left[\begin{array}{cccc} X[1] & X[2] & X[3] & X[4] \end{array}\right]\in M_{100\times (4\cdot 90003)}\left(R\right)=M_{100\times 360012}\left(R\right). \end{array}$$

If matrix X[1,2,3,4] in Equation (18) is used for the damage detection and classification procedure introduced in Section 3 and Section 4, which this allows analyzation of the information of all the actuation phases at one time, a single classification is obtained that combines these four phases. Finally, we can also use the separate classification obtained for each actuation phase so that each actuation phase casts a vote thus leading to a final decision based on the four actuation phases. These strategies will be explained in detail in Section 5.5.

### 5.4. κ−Fold Non-Exhaustive Leave-p-Out Cross Validation

The analysis of the proposed approach is done by comparing *test* data, i.e., the new experiments in *unknown* state under the same conditions, with *baseline* data, which is data from the structure under different structural states. To this end, we use the κ−fold non-exhaustive leave-*p*-out cross validation described in the subsequent paragraphs.

For the sake of clarity, let us write X[Φ] to refer to both matrix X[φ] in Equation (17) and matrix X[1,2,3,4] in Equation (18). Some of the rows in X[Φ] will be used as the baseline data to build the model and the clusters, i.e., υ=5 rows per structural state, and the rest of the rows are used for the validation. More precisely, we will perform five iterations (κ=5) of a non-exhaustive leave-*p*-out cross validation, where $p=\sum_{i=1}^{E}\left(n_{i}-\upsilon \right)=n_{1}+n_{2}+n_{3}+n_{4}-\upsilon \cdot E=80$, to estimate the overall accuracy and avoid overfitting. Let us define, for each structural state l=1,…,E, the permutation $\sigma_{l}$:

$$\begin{array}{cc} \sigma_{l}:\left\{1,2,\ldots ,n_{l}\right\} & \to \{1,2,\ldots ,n_{l}\}\\ i & \to \sigma_{l}\left(i\right) \end{array}$$

In this particular case, $n_{1}=n_{2}=n_{3}=n_{4}=25$. Therefore, in the first iteration, the baseline data to build the model are the matrix:

(19) $$\begin{array}{cc} X & =S^{⊤}\cdot X\left[\Phi \right], \end{array}$$

(20) $$\begin{array}{ll} S & =[\begin{array}{cccccccccc} e_{\sigma_{1}\left(1\right)} & \cdots & e_{\sigma_{1}\left(5\right)} & e_{n_{1}+\sigma_{2}\left(1\right)} & \cdots & e_{n_{1}+\sigma_{2}\left(5\right)} & \cdots & e_{n_{1}+n_{2}+n_{3}+\sigma_{4}\left(1\right)} & \cdots & e_{n_{1}+n_{2}+n_{3}+\sigma_{4}\left(5\right)} \end{array}], \end{array}$$

where $e_{j}\in R^{n_{1}+n_{2}+n_{3}+n_{4}}=R^{100}$ is the *j*-th element of the canonical basis of the real vector space $R^{n_{1}+n_{2}+n_{3}+n_{4}}=R^{100}$, and $S\in M_{\left(n_{1}+n_{2}+n_{3}+n_{4}\right)\times \left(\nu \cdot E\right)}\left(R\right)$ is the *selector* matrix. Basically, matrix X in Equation (19) has been built by randomly selecting υ=5 rows per structural state. The $\sum_{i=1}^{E}\left(n_{i}-\upsilon \right)=80$ rows of matrix X[Φ] that are not used to build the model are used for the validation.

In the *i*-th iteration, i=1,…,κ, the baseline data to build the model are the matrix:

$$\begin{array}{lll} X & = & S^{⊤}\cdot X\left[\Phi \right],\\ S & = & \left[\begin{array}{ccccccc} e_{\sigma_{1}\left(5\left(i-1\right)+1\right)} & \cdots & e_{\sigma_{1}\left(5\left(i-1\right)+5\right)}| & e_{n_{1}+\sigma_{2}\left(5\left(i-1\right)+1\right)} & \cdots & e_{n_{1}+\sigma_{2}\left(5\left(i-1\right)+5\right)}| & \cdots \end{array}\right.\\ & & \left.\begin{array}{cccc} \cdots | & e_{n_{1}+n_{2}+n_{3}+\sigma_{4}\left(5\left(i-1\right)+1\right)} & \cdots & e_{n_{1}+n_{2}+n_{3}+\sigma_{4}\left(5\left(i-1\right)+5\right)} \end{array}\right] \end{array}$$

where, as in Equation (19), $e_{j}\in R^{n_{1}+n_{2}+n_{3}+n_{4}}=R^{100}$ is the *j*-th element of the canonical basis of the real vector space $R^{n_{1}+n_{2}+n_{3}+n_{4}}=R^{100}$ and, as in Equation (20), $S\in M_{\left(n_{1}+n_{2}+n_{3}+n_{4}\right)\times \left(\nu \cdot E\right)}\left(R\right)$ is the *selector* matrix. Since $\sum_{i=1}^{E}\left(n_{i}-\upsilon \right)=80$ rows of matrix X[Φ] will be used for the validation step and with respect to κ=5 iterations, the sum of all the elements in the *confusion matrices* that we will present in Section 6 is equal to $\left(\sum_{i=1}^{E}\left(n_{i}-\upsilon \right)\right)\cdot \kappa =400$.

### 5.5. Application of the Damage Detection and Classification Procedure

In this section, two strategies are presented to apply the damage detection and classification procedure. These two strategies are:

(1) the classification is based on a single matrix: X[1],X[2],X[3],X[4] or X[1,2,3,4], as defined in Equations (17) and (18), respectively, with κ−fold non-exhaustive leave-*p*-out cross validation;
(2) the classification is based on the four matrices X[1],X[2],X[3] and X[4], defined in Equation (17), with respect to the four actuation phases, with κ−fold non-exhaustive leave-*p*-out cross validation. Each actuation phase will cast a vote and a final decision is taken.

In the first case, in a succinct way, the following seven steps are performed:

- **Step 1**. The data in matrix X are scaled using MCGS to define a new matrix $\overset{˘}{X}$.
- **Step 2**. PCA is applied to $\overset{˘}{X}$ to obtain the PCA model P.
- **Step 3**. The number ℓ=D∈N of principal components is chosen so that the proportion of variance explained is greater than or equal to 95%. Therefore, the reduced PCA model is $P_{\ell }$.
- **Step 4**. A realization $z^{⊤}\in R^{3\cdot 30001}=R^{90003}$ —for X[1],X[2],X[3] and X[4]— or $z^{⊤}\in R^{4\cdot 90003}=R^{360012}$ —for X[1,2,3,4]— of the current structure to diagnose is needed. Then, vector $z^{⊤}$ is scaled as in Equation (14) to define ${\overset{˘}{z}}^{⊤}$.
- **Step 5**. The data points set X is defined as:
  $$\begin{array}{c} X^{\prime }=\left\{x^{1},\ldots ,x^{20},x^{21}\right\}\subset R^{\ell }, \end{array}$$
  where
  $$\begin{array}{cc} x^{i} & =e_{i}^{⊤}\overset{˘}{X}P_{\ell },\;i=1,\ldots ,20,\\ x^{21} & ={\overset{˘}{z}}^{⊤}P_{\ell }. \end{array}$$
  Subsequently, *t*-SNE is applied to this ℓ−dimensional data set $X^{\prime }$ to find a collection of 2−dimensional map points:
  $$\begin{array}{c} Y^{\prime }=\left\{y^{1},\ldots ,y^{20},y^{21}\right\}\subset R^{2}. \end{array}$$
- **Step 6**. E=4 clusters are obtained, that are related to the E=4 different structural states. These clusters are formed by the map points:
  $$\begin{array}{cc} \left\{y^{1},\ldots ,y^{5}\right\} & \subset Y,\mathrm{related}\;\mathrm{to}\;\mathrm{the}\;1st\;\mathrm{structural}\;\mathrm{state};\\ \left\{y^{6},\ldots ,y^{10}\right\} & \subset Y,\mathrm{related}\;\mathrm{to}\;\mathrm{the}\;2nd\;\mathrm{structural}\;\mathrm{state};\\ \left\{y^{11},\ldots ,y^{15}\right\} & \subset Y,\mathrm{related}\;\mathrm{to}\;\mathrm{the}\;3rd\;\mathrm{structural}\;\mathrm{state};\\ \left\{y^{16},\ldots ,y^{20}\right\} & \subset Y,\mathrm{related}\;\mathrm{to}\;\mathrm{the}\;4th\;\mathrm{structural}\;\mathrm{state}. \end{array}$$
  The centroid $Y_{l},\;l=1,\ldots ,E$, associated with the *l*-th structural state is computed as in Equation (16).
- **Step 7**. Finally, the current structure to diagnose is associated to the *l*-th structural state if
  $$\begin{array}{c} l=\arg\min_{l=1,\ldots ,E}{∥Y_{l}-y^{21}∥}_{2}. \end{array}$$

**Figure 6:**
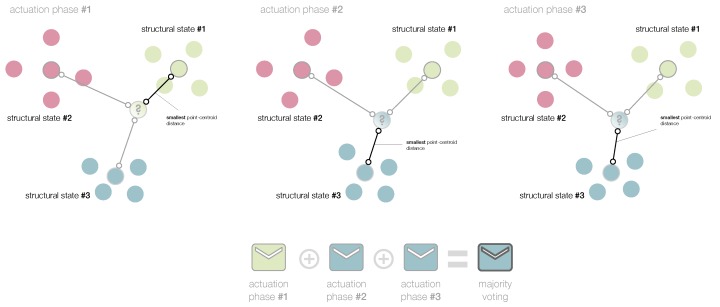
In the majority voting, the strategy of the smallest point-centroid distance is performed per actuation phase. The current structure to diagnose is associated to the most voted structural state.

In the second case, we follow **Step 1** to **Step 6** above for the four matrices X[φ],φ=1,…,4, related to the four actuation phases. With the information provided by the four actuation phases, several approaches can be considered to finally classify the structure that has to be diagnosed. One of these approaches, majority voting, is widely used in standard fusion schemes [28], as well as weighted majority vote or soft voting. For our case of the small aluminum plate, the majority voting will be used, as well as an approach based on the sum of the inverse distances between the centroids and the map point, which is somehow related to a weighted majority vote. Here are the details of both approaches:

- **Majority voting**. In this case, the strategy of the smallest point-centroid distance is performed four times, one per actuation phase. Therefore, four classifications are obtained for a single structure to diagnose. More precisely, each actuation phase acts as a *classifier*. Figure 6 illustrates this idea with respect to three actuation phases.
  The current structure to diagnose, in the φ-th actuation phase, φ=1,…,4, is associated to the $l_{\varphi }$-th structural state if
  $$\begin{array}{c} l_{\varphi }=\arg\min_{l=1,\ldots ,E}{∥Y_{l}^{\varphi }-y_{\varphi }^{21}∥}_{2}. \end{array}$$
  It is worth remembering that $y_{\varphi }^{21}\in R^{2}$ is the map point associated to the realization of the current structure to diagnose. The structure is finally classified according to the most repeated classification. That is, the current structure to diagnose is associated to the *l*-th structural state if
  $$\begin{array}{c} l=\mathrm{mode}\{l_{1},l_{2},l_{3},l_{4}\}, \end{array}$$
  in the case of a unimodal set. In the case of a bi-modal set, if the two modal values are $l_{\alpha }$ and $l_{\beta }$, the current structure to diagnose is associated to the *l*-th structural state if
  $$\begin{array}{c} l=\arg\min_{l\in \{l_{\alpha },l_{\beta }\}}\sum_{\varphi =1}^{4}{∥Y_{l}^{\varphi }-y_{\varphi }^{21}∥}_{2}. \end{array}$$
  Finally, if the set $\left\{l_{1},l_{2},l_{3},l_{4}\right\}$ is a set with no mode, the structure is associated to the *l*-th structural state if
  $$\begin{array}{c} l=\arg\min_{l=1,\ldots ,E}\sum_{\varphi =1}^{4}{∥Y_{l}^{\varphi }-y_{\varphi }^{21}∥}_{2}. \end{array}$$
- **Sum of the inverse distances**. In this case, for a given structural state, we sum the inverse of the distances between the centroids $Y_{l}^{\varphi }$ and the map point $y_{\varphi }^{21}$, for all the actuation phases φ=1,…,4. The assigned structural state is the one that obtains the highest sum. More precisely, the current structure to diagnose is associated to the *l*-th structural state if
  $$\begin{array}{c} l=\arg\max_{l=1,\ldots ,E}\sum_{\varphi =1}^{4}\frac{1}{∥Y_{l}^{\varphi }-y_{\varphi }^{21}{∥}_{2}}. \end{array}$$
  It is worth remarking that the arguments of the maxima of the sum of the inverse distances is equivalent to the arguments of the minima of the harmonic mean of these distances. More precisely, for a given structural state, the harmonic mean of the distances between the centroids $Y_{l}^{\varphi }$ and the map point $y_{\varphi }^{21}$, for all the actuation phases φ=1,…,4 is
  $$\begin{array}{c} \frac{1}{\frac{1}{4}\sum_{\varphi =1}^{4}\frac{1}{∥Y_{l}^{\varphi }-y_{\varphi }^{21}{∥}_{2}}}. \end{array}$$
  Therefore,
  $$\begin{array}{cc} l & =\arg\max_{l=1,\ldots ,E}\sum_{\varphi =1}^{4}\frac{1}{∥Y_{l}^{\varphi }-y_{\varphi }^{21}{∥}_{2}}=\arg\min_{l=1,\ldots ,E}\frac{1}{\frac{1}{4}\sum_{\varphi =1}^{4}\frac{1}{∥Y_{l}^{\varphi }-y_{\varphi }^{21}{∥}_{2}}}. \end{array}$$
  S. Mehta et al. [29] also uses the harmonic distance to define a pattern classification technique similar to *k*-nearest neighbors classifier.

## 6. Results

In this section, the results of the application of the damage detection and classification procedure, introduced in Section 3 and Section 4 and detailed in Section 5.3, Section 5.4 and Section 5.5, to the aluminum plate are presented in terms of the confusion matrices and with respect to the scenarios defined in Section 5.2. The results for each scenario are presented in a different section. More precisely, in Section 6.1 the results with respect to **Scenario 1** are presented. Equivalently, Section 6.2 and Section 6.3 present the results with respect to **Scenario 2** and **Scenario 3**, respectively. In the three scenarios, four different structural states have been considered:

- the first structural state corresponds to the healthy state of the structure, that is, the square aluminum plate with no damage, noted as D0;
- the second, third, and fourth structural states correspond to the plate with an added mass at the positions indicated in Figure 3 and Figure 5, noted as D1, D2, and D3, respectively.

To validate the damage detection and classification detailed in Section 5.3, Section 5.4 and Section 5.5, we will perform five iterations (κ=5) of a non-exhaustive leave-*p*-out cross validation, where p=80, as described in Section 5.4. At each iteration, a total of 80 realizations have been considered, according to the following distribution: 20 realization per structural state (D0,D1,D2, and D3). Since 80 realizations have been used for the validation step and with respect to κ=5 iterations, the sum of all the elements in the confusion matrices that we will present in Section 6.1, Section 6.2 and Section 6.3 is equal to 5·80=400.

Again, for the three scenarios, seven different confusion matrices are presented:

- **Actuation phase 1**. The damage detection and classification procedure is applied to a single matrix, X[1], as in Equation (17), using the smallest point-centroid distance.
- **Actuation phase 2**. The damage detection and classification procedure is applied to a single matrix, X[2], as in Equation (17), using the smallest point-centroid distance.
- **Actuation phase 3**. The damage detection and classification procedure is applied to a single matrix, X[3], as in Equation (17), using the smallest point-centroid distance.
- **Actuation phase 4**. The damage detection and classification procedure is applied to a single matrix, X[4], as in Equation (17), using the smallest point-centroid distance.
- **Actuation phases 1–4**. The damage detection and classification procedure is applied to a single matrix, i.e., the horizontal concatenation of the four matrices X[φ],φ=1,…,4, X[1,2,3,4], as in Equation (18), using the smallest point-centroid distance.
- **Majority voting**. The damage detection and classification procedure is applied to the four X[φ],φ=1,…,4. Each actuation phase casts a vote, and a final decision is taken based on majority voting (Section 5.5).
- **Sum of the inverse distances**. The damage detection and classification procedure is applied to the four X[φ],φ=1,…,4. Each actuation phase casts a vote and a final decision is taken based on the maximum sum of the inverse distances (Section 5.5).

Finally, in some cases, and with the purpose of comparing the performance of the current damage detection and classification approach, confusion matrices in the frequency and time domains have been included.

**Figure 7:**
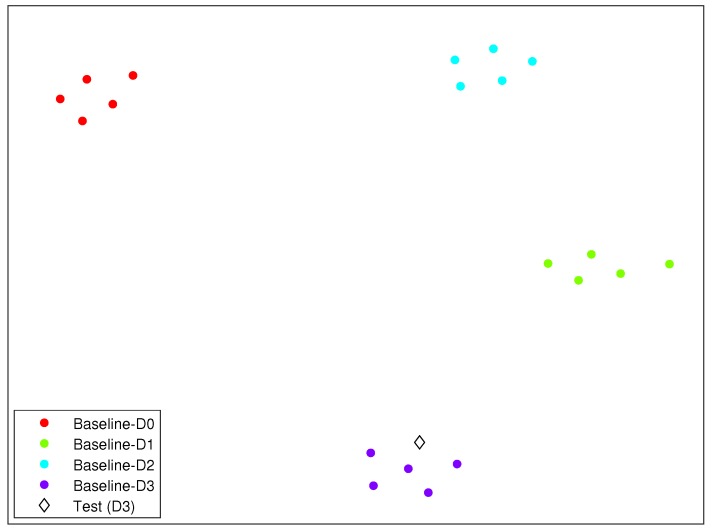
Clusters formed by the different structural states described in Section 6, for Scenario 3. The diamond represents the structure to diagnose.

As an illustrating example, we have included in Figure 7 the clusters formed by the different structural states described in this section and in the case of Scenario 3. In this figure, the diamond represents the structure to diagnose. It can be clearly observed how the diamond is close to the cluster related to damage 3.

### 6.1. Scenario 1

In this section, the results with respect to **Scenario 1** are presented. It is worth noting that, in this scenario, a short wire has been used, and the measured signals are filtered with a SG algorithm. The seven confusion matrices can be found in Table 1 and Table 2. When the decision is based on a single actuation phase (Table 1), the overall accuracy is quite good. More precisely, with 397 in the actuation phase 1, 399 in the actuation phase 2, 395 in the actuation phase 3, and 397 in the actuation phase 4, realizations have been correctly classified out of 400 cases, which represents an overall accuracy of 99.25%, 99.75%, 98.75%, and 99.25%, respectively. When the four actuation phases are used at the same time (actuation phases 1–4, Equation (18), majority voting, and sum of the inverse distances), an overall accuracy of 99–100% is achieved, as it can be observed from Table 2.

### 6.2. Scenario 2

In this section, the results with respect to **Scenario 2** are presented. In this case, a short wire has been used but the measured signals are not filtered. The seven confusion matrices can be found in Table 3 and Table 4. When the decision is based on a single actuation phase (Table 3), the overall accuracy is very remarkable. More precisely, with respect to actuation phase 1, 2, and 3, 400 realizations have been correctly classified out of 400 cases, which represents an overall accuracy of 100%. With respect to actuation phase 4, 399 realizations have been correctly classified out of 400 cases, that is to say, an overall accuracy of 99.75%. When the four actuation phases are used at the same time (actuation phases 1–4, Equation (18), majority voting, and sum of the inverse distances), an overall accuracy of 100% is achieved, as it can be observed from Table 4.

### 6.3. Scenario 3

The results with respect to **Scenario 3** are finally presented in this section. In the two previous scenarios, a short wire was used. However, in this case, the signals are acquired using a 2.5 m long wire. Table 5 and Table 6 include the seven confusion matrices. When the decision is based on a single actuation phase (Table 5), the overall accuracy is quite good, too. More precisely, with 384 in the actuation phase 1, 395 in the actuation phase 2, 395 in the actuation phase 3, and 398 in the actuation phase 4, realizations have been correctly classified, which represents an overall accuracy of 96%, 98.75%, 98.75%, and 99.5%, respectively. When the four actuation phases are used at the same time (actuation phases 1–4, Equation (18), majority voting, and sum of the inverse distances), an overall accuracy of 99.5–100% is achieved, as it can be observed from Table 6.

The potential of the approaches where the four actuation phases are used can be observed in this last scenario, see Table 6:

- When the four actuation phases are merged in a single matrix as in Equation (18), 398 realizations have been correctly classified out of 400 cases, which represents an overall accuracy of 99.5%.
- When each actuation phase casts a vote and a final decision is taken based on majority voting, the overall accuracy is increased to 100%.
- Finally, when each actuation phase casts a vote and a final decision is taken based on the maximum sum of the inverse distances, the overall accuracy is increased to 100%, too.

In addition, in this scenario, the results in the frequency domain are compared to those in the time domain [11]. In the time domain, when the decision is based on a single actuation phase (Table 7), with 244 in the actuation phase 1, 398 in the actuation phase 2, 280 in the actuation phase 3, and 277 in the actuation phase 4, realizations have been correctly classified out of 400 cases. This represents an overall accuracy of 61%, 99.5%, 70%, and 69.25%, respectively. Clearly, the strategy in the frequency domain, i.e., the overall accuracy fluctuates between 96% and 99.5%, outperforms the approach in the time domain. In addition, the false positive rate (FPR), i.e., the number of false positives with respect to the total number of negatives, and the false negative rate (FNR), i.e., the number of false negatives with respect to the total number of positives, are clearly unsatisfactory in the time domain. However, FPR and FNR are significantly reduced to values close to 0% in the frequency domain. It is worth noting that in the computation of the FNR, the three different types of damage D1,D2, and D3 are considered as a single category, just the opposite of the healthy state of the structure. In the time domain, when the four actuation phases are used at the same time (Table 8), the overall accuracy is of 88.5% in actuation phases 1–4, of 91.25% in majority voting, and of 97% in sum of the inverse distances, whereas the overall accuracy is increased to 99.5%, 100%, and 100%, respectively, in the frequency domain. At the same time, FPR and FNR are reduced to 0% in the frequency domain, while they are slightly increased in the time domain. All this is an indication of the better quality of the clusters created in the frequency domain than the ones created in the time domain. Table 9 summarizes the values for the overall accuracy, the FPR, and the FNR in this scenario and in the time and frequency domains.

### 6.4. General Comments

The results presented in Section 6.1, Section 6.2 and Section 6.3 reveal that it is better to make a decision considering all of the actuation phases (assembling theses phases or using them to cast a vote) rather than working with the phases separately. On the other hand, the results also show that both strategies majority voting and sum of the inverse distances slightly outperform the horizontal concatenation of the four actuation phases in the frequency domain. However, in the time domain (**Scenario 3**, Table 8), the results reveal (i) the strong performance of the sum of the inverse distances strategy, which clearly classifies the practical totality of the kinds of damage that we have considered, compared to majority voting or the horizontal concatenation of the four actuation phases; and (ii) that the majority voting outperforms the horizontal concatenation of the four actuation phases, but it cannot completely classify damage D1.

It is worth noting that, in general, the healthy state of the structure is confused with the structure with damage in just a few cases. Similarly, the structure with damage is identified as a structure with no damage in a very limited number of realizations.

In general, the performance of the proposed methodology is very satisfactory when the signals are acquired using a short wire, with or without adding white Gaussian noise. In these two cases, using PCA as a pre-processing step, the noise is canceled. The third scenario presents the worst case because it used a long cable (2.5 m) from the digitizers to the sensors. In this scenario, the signals were badly digitized due to the impedance of the cable, the low voltage of the stimulus, and other experimental features. Therefore, it can be observed that the use of a long cable from the digitizer to the sensors affects in the detection and classification method. However, combining the four actuation phases with

(i) the sum of the inverse distances strategy, in the time domain; or
(ii) the majority voting strategy or the sum of the inverse distances strategy, in the frequency domain,

very accurate results can be obtained.

It should be noted that, in general, it is better to work in the frequency domain than in the time domain because the obtained results are significantly improved, as it can be clearly observed in the third scenario.

## 7. Conclusions

In this work, a SHM strategy for detection and classification of structural changes based on a two-step data integration (type *E* unfolding and MCGS), data transformation using PCA, and a two-step data reduction combining PCA and *t*-SNE has been proposed. The proposed approach is evaluated using experimental data. In general, the results obtained show that the performance of the proposed methodology is very satisfactory, given its high classification accuracy; and its behavior is very good and similar in all the data sets.

In the case study, very accurate results are obtained with or without adding white Gaussian noise, since PCA cancels the noise. However, the use of a long wire (2.5 m) from the digitizers to the sensors negatively affects the detection and classification method. But combining the four actuation phases with the sum of the inverse distances strategy, in the time domain, and with the majority voting strategy or the sum of the inverse distances strategy, in the frequency domain, accurate results can be obtained. Results also show that the quality of the two-dimensional clusters created with *t*-SNE in the frequency domain is better than the quality of the two-dimensional clusters created with *t*-SNE in the time domain, thus leading to a better classification. Therefore, the strategy in the frequency domain significantly outperforms the approach in the time domain.

Some aspects to highlight in the proposed methodology are: the *t*-SNE technique has been extended and adapted to the field of SHM, in the detection and classification of structural changes; the method classifies the current state of the structure by means of a data-driven analysis, that is, using collected data from the structure under different structural states and without the use of complex mathematical models; it is better to make a decision considering all of the actuation phases (assembling theses phases or using them to cast a vote) rather than working with the phases separately; both strategies, majority voting and sum of the inverse distances, slightly outperform the horizontal concatenation of the four actuation phases in the frequency domain; in the time domain, sum of the inverse distances strategy outperforms majority voting, and this last strategy outperforms the horizontal concatenation of the four actuation phases; it is better to work in the frequency domain than in the time domain because better results are obtained; and finally, in general, the healthy state of the structure is confused with the structure with damage in just a few cases, and similarly, the structure with damage is identified as a structure with no damage in a very limited number of realizations. With respect to the possible fields of application, similar aluminum plates have been used to represent parts of a plane (wings or fuselage). We think that we can also apply this approach for the damage and fault detection of wind turbines. In general, there is no a prescribed field of application: if a sensor network can be installed in a structure, and several actuation phases can be considered, the proposed approach can be implemented a priori.

As a future work, we plan to develop further the proposed method for different EOC to determine its effectiveness, as well as to handle imbalanced data. In addition, we aim to investigate the parametric version of *t*-SNE. 

## Figures and Tables

**Table 1:** Confusion matrix of the application of the *t*-SNE based damage detection and classification procedure presented in Section 3 and Section 4 to the case of the aluminum plate in **Scenario 1**, in the **frequency domain**. Rows represent true values, while columns represent predicted values.

	Actuation Phase 1				Actuation Phase 2				Actuation Phase 3				Actuation Phase 4			
	D0	D1	D2	D3	D0	D1	D2	D3	D0	D1	D2	D3	D0	D1	D2	D3
D0	97	0	2	1	100	0	0	0	100	0	0	0	100	0	0	0
D1	0	100	0	0	0	100	0	0	0	100	0	0	0	100	0	0
D2	0	0	100	0	0	0	100	0	3	0	96	1	0	0	98	2
D3	0	0	0	100	0	0	1	99	1	0	0	99	0	0	1	99

D0 (healthy state of the structure); D1,D2, and D3 (added masses at the positions indicated in Figure 3 and Figure 5).

**Table 2:** Confusion matrix of the application of the *t*-SNE based damage detection and classification procedure presented in Section 3 and Section 4 to the case of the aluminum plate in **Scenario 1**, when the four actuation phases are used at the same time, in the **frequency domain**. Rows represent true values, while columns represent predicted values.

	Phases 1–4				Majority Voting				Inverse Distances			
	D0	D1	D2	D3	D0	D1	D2	D3	D0	D1	D2	D3
D0	100	0	0	0	100	0	0	0	100	0	0	0
D1	0	99	1	0	0	100	0	0	0	100	0	0
D2	1	0	99	0	0	0	100	0	0	0	100	0
D3	0	0	0	100	0	0	0	100	0	0	0	100

D0 (healthy state of the structure); D1,D2, and D3 (added masses at the positions indicated in Figure 3 and Figure 5).

**Table 3:** Confusion matrix of the application of the *t*-SNE based damage detection and classification procedure presented in Section 3 and Section 4 to the case of the aluminum plate in **Scenario 2**, in the **frequency domain**. Rows represent true values, while columns represent predicted values.

	Actuation Phase 1				Actuation Phase 2				Actuation Phase 3				Actuation Phase 4			
	D0	D1	D2	D3	D0	D1	D2	D3	D0	D1	D2	D3	D0	D1	D2	D3
D0	100	0	0	0	100	0	0	0	100	0	0	0	100	0	0	0
D1	0	100	0	0	0	100	0	0	0	100	0	0	0	100	0	0
D2	0	0	100	0	0	0	100	0	0	0	100	0	0	0	100	0
D3	0	0	0	100	0	0	0	100	0	0	0	100	0	0	1	99

D0 (healthy state of the structure); D1,D2, and D3 (added masses at the positions indicated in Figure 3 and Figure 5).

**Table 4:** Confusion matrix of the application of the *t*-SNE based damage detection and classification procedure presented in Section 3 and Section 4 to the case of the aluminum plate in **Scenario 2**, when the four actuation phases are used at the same time, in the **frequency domain**. Rows represent true values, while columns represent predicted values.

	Phases 1–4				Majority Voting				Inverse Distances			
	D0	D1	D2	D3	D0	D1	D2	D3	D0	D1	D2	D3
D0	100	0	0	0	100	0	0	0	100	0	0	0
D1	0	100	0	0	0	100	0	0	0	100	0	0
D2	0	0	100	0	0	0	100	0	0	0	100	0
D3	0	0	0	100	0	0	0	100	0	0	0	100

D0 (healthy state of the structure); D1,D2, and D3 (added masses at the positions indicated in Figure 3 and Figure 5).

**Table 5:** Confusion matrix of the application of the *t*-SNE based damage detection and classification procedure presented in Section 3 and Section 4 to the case of the aluminum plate in **Scenario 3**, in the **frequency domain**. Rows represent true values, while columns represent predicted values.

	Actuation Phase 1				Actuation Phase 2				Actuation Phase 3				Actuation Phase 4			
	D0	D1	D2	D3	D0	D1	D2	D3	D0	D1	D2	D3	D0	D1	D2	D3
D0	98	2	0	0	99	0	1	0	100	0	0	0	100	0	0	0
D1	6	90	2	2	0	99	1	0	0	95	5	0	1	99	0	0
D2	1	1	97	1	0	1	97	2	0	0	100	0	0	0	100	0
D3	0	1	0	99	0	0	0	100	0	0	0	100	0	0	1	99

D0 (healthy state of the structure); D1,D2, and D3 (added masses at the positions indicated in Figure 3 and Figure 5).

**Table 6:** Confusion matrix of the application of the *t*-SNE based damage detection and classification procedure presented in Section 3 and Section 4 to the case of the aluminum plate in **Scenario 3**, when the four actuation phases are used at the same time, in the **frequency domain**. Rows represent true values, while columns represent predicted values.

	Phases 1–4				Majority Voting				Inverse Distances			
	D0	D1	D2	D3	D0	D1	D2	D3	D0	D1	D2	D3
D0	100	0	0	0	100	0	0	0	100	0	0	0
D1	0	99	0	1	0	100	0	0	0	100	0	0
D2	0	1	99	0	0	0	100	0	0	0	100	0
D3	0	0	0	100	0	0	0	100	0	0	0	100

D0 (healthy state of the structure); D1,D2, and D3 (added masses at the positions indicated in Figure 3 and Figure 5).

**Table 7:** Confusion matrix of the application of the *t*-SNE based damage detection and classification procedure presented in Section 3 and Section 4 to the case of the aluminum plate in textbfScenario 3, in the **time domain**. Rows represent true values, while columns represent predicted values.

	Actuation Phase 1				Actuation Phase 2				Actuation Phase 3				Actuation Phase 4			
	D0	D1	D2	D3	D0	D1	D2	D3	D0	D1	D2	D3	D0	D1	D2	D3
D0	50	6	19	25	100	0	0	0	93	1	3	3	53	21	1	25
D1	19	66	11	4	0	100	0	0	16	50	23	11	17	61	2	20
D2	14	3	73	10	0	1	98	1	5	19	70	6	5	3	76	16
D3	15	9	21	55	0	0	0	100	0	23	10	67	10	3	0	87

D0 (healthy state of the structure); D1,D2, and D3 (added masses at the positions indicated in Figure 3 and Figure 5).

**Table 8:** Confusion matrix of the application of the *t*-SNE based damage detection and classification procedure presented in Section 3 and Section 4 to the case of the aluminum plate in **Scenario 3**, when the four actuation phases are used at the same time, in the **time domain**. Rows represent true values, while columns represent predicted values.

	Phases 1–4				Majority voting				Inverse distances			
	D0	D1	D2	D3	D0	D1	D2	D3	D0	D1	D2	D3
D0	86	1	7	6	98	0	0	2	99	0	1	0
D1	8	88	4	0	12	85	2	1	1	99	0	0
D2	1	8	89	2	6	2	90	2	1	1	95	3
D3	3	4	2	91	0	3	5	92	1	2	2	95

D0 (healthy state of the structure); D1,D2, and D3 (added masses at the positions indicated in Figure 3 and Figure 5).

**Table 9:** Overall accuracy, false positive rate (FPR) and false negative rate (FNR) of the application of the *t*-SNE-based damage detection and classification procedure presented in Section 3 and Section 4 to the case of the aluminum plate in **Scenario 3**, when the four actuation phases are used separately and at the same time, in both domains, time and frequency.

	Accuracy		FPR		FNR	
	Time	Frequency	Time	Frequency	Time	Frequency
Actuation phase 1	61.0%	96.0%	50.0%	2.0%	16.0%	2.3%
Actuation phase 2	99.5%	98.8%	0.0%	1.0%	0.0%	0.0%
Actuation phase 3	70.0%	98.8%	7.0%	0.0%	7.0%	0.0%
Actuation phase 4	69.3%	99.5%	47.0%	0.0%	10.7%	0.3%
Phases 1-4	88.5%	99.5%	14.0%	0.0%	4.0%	0.0%
Majority voting	91.3%	100.0%	2.0%	0.0%	6.0%	0.0%
Inverse distances	97.0%	100.0%	1.0%	0.0%	1.0%	0.0%

## Nomenclature

X	High-dimensional data set.
$X^{\prime }$	High-dimensional data set including the data point to diagnose.
Y	Low-dimensional map points.
$Y^{\prime }$	Low-dimensional map points including the map point to diagnose.
$Y_{l}$	Centroid associated with the *l*-th structural state.
P	Similarity matrix for the high-dimensional data points.
Q	Similarity matrix for the low-dimensional map points.
C	KL divergence.
η	Learning rate.
α(t)	Momentum term at iteration *t*.
*p*	Perplexity.
X	Matrix that collects all the realizations under different structural states.
$\overset{˘}{X}$	Scaled original —in the frequency domain— data matrix.
*N*	Number of sensors.
*L*	Number of components in each signal.
*E*	Number of different structural states.
$C_{\overset{˘}{X}}$	Covariance matrix of $\overset{˘}{X}$.
$\lambda_{k}$	Eigenvalues.
$\rho_{k}$	Eigenvectors.
Λ	Eigenvalues in a diagonal matrix.
P	PCA model (full case).
$P_{\ell }$	PCA model (reduced).
T	Projection of the scaled data set $\overset{˘}{X}$ into the subspace spanned by the PCA model.
$e_{i}$	*i*-th element of the canonical basis.
z	Current original —in the frequency domain— data vector to diagnose.
$\overset{˘}{z}$	Current scaled original —in the frequency domain— data vector to diagnose.
φ	φ-th actuation phase.
S	Selector matrix

## Abbreviations

CM	Condition monitoring
EOC	Environmental and operational conditions
FFT	Fast Fourier transform
FNR	False negative rate
FPR	False positive rate
KL	Kullback-Leibler
MCGS	Mean-centered group scaling
PCA	Principal component analysis

PZT	Piezoelectric transducer
SG	Savitzky–Golay
SHM	Structural health monitoring
SNE	Stochastic neighbor embedding
*t*-SNE	*t*-distributed stochastic neighbor embedding
